# Influenza A virus RNA localisation and the interceding trafficking pathways of the host cell

**DOI:** 10.1371/journal.ppat.1013090

**Published:** 2025-04-23

**Authors:** Stefano Bonazza, David G. Courtney

**Affiliations:** Wellcome-Wolfson Institute for Experimental Medicine, Queen’s University Belfast, Belfast, United Kingdom; Boston University, UNITED STATES OF AMERICA

## Abstract

Viruses have evolved to efficiently navigate host cells to deliver, express, and replicate their genetic material. Understanding the mechanisms underlying viral RNA localisation is paramount to designing new antivirals. In this review, we discuss Influenza A Virus (IAV) as a model system to highlight some of the ways in which RNA viruses can hijack the endomembrane systems, as well as nuclear transporters, to achieve the correct localisation of their transcripts. IAV exemplifies a nuclear-replicating RNA virus with a complex and highly regulated RNA localisation and trafficking system within host cells. The virus subverts various vesicular transport systems and nuclear transporters, altering normal cellular functions. IAV RNA trafficking begins during entry; after clathrin-mediated endocytosis, the viral genome (vRNPs) is released into the cytosol after fusion with the endosomal membrane, and it is subsequently imported into the nucleus via the importin system. There, vRNPs engage with most major subnuclear structures and exploit host chromatin, the transcription machinery and splicing apparatus to achieve efficient viral mRNA synthesis and export. Subsequently, newly synthesised vRNPs are rapidly exported from the nucleus and contact the host’s recycling endosome network for transport to the plasma membrane. We discuss the critical viral remodelling of the entire endomembrane system, particularly the Rab11 recycling endosome and the endoplasmic reticulum. Lastly, replicated genomes come together into bundles to be inserted in budding virions, and we discuss the current models being proposed and the evidence behind them. Despite advances in understanding these processes, several knowledge gaps remain, particularly regarding the specific export of unspliced IAV transcripts, the remodelling of the endomembrane system, and segment bundling.

## 1. Introduction

Viruses are self-replicating gene delivery systems, optimised to transport their genetic information to cells, where they subvert the host machinery to generate copies of themselves. As such, trafficking of genetic material is a key function of all viruses. Developing a more thorough understanding of how viruses exploit these mechanisms would not only greatly enhance our development of antivirals, but also our overall knowledge of a fundamental aspect of cell biology.

Given its status as a nuclear-replicating RNA virus, influenza A virus (IAV) is an ideal pathogen to model viral intracellular RNA trafficking. Indeed, IAV RNAs are constantly transported from the nucleus to the cytosol and vice versa throughout the viral replication cycle in a very efficient and regulated manner. This review aims to detail the current knowledge, models, and open questions surrounding the field of IAV RNA trafficking.

### 1.1. Influenza A virus

IAV is a member of the Orthomyxovirus family of viruses, and it is a major human and animal pathogen. It is estimated that IAV alone is responsible for up to 500,000 annual deaths [1], mainly arising during annual seasonal outbreaks. Due to its segmented genomic structure, ample and promiscuous animal reservoir, and coinfection potential, influenza underwent several zoonotic spillover events in the past century, giving rise to four human pandemics thus far (discussed further in [2]).

**Fig 1:**
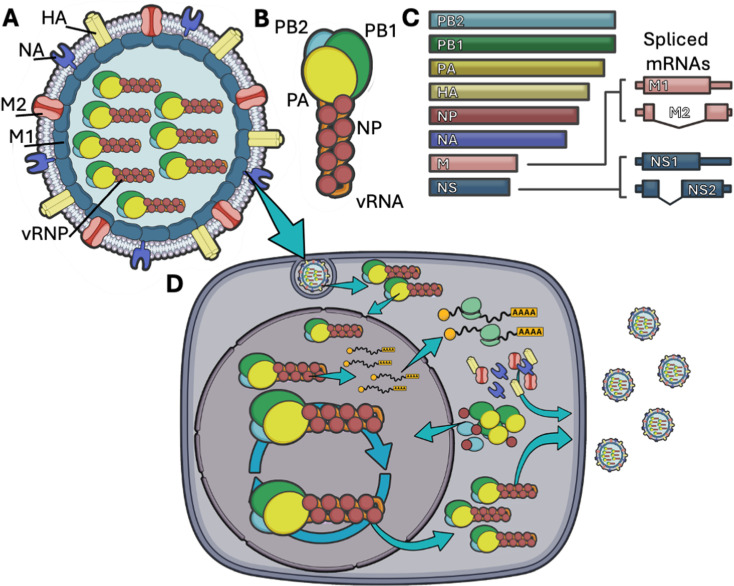
Influenza A Virus structure, genome, and replication cycle. **(A)** Schematic representation of the IAV virion. HA, NA, and M2 are included in the lipid envelope, oligomeric M1 provides structure to the internal membrane leaflet, while the eight viral ribonucleoparticles (vRNPs) reside in the inner core. **(B)** Organisation of the vRNP. The trimeric influenza RNA polymerase (FluPol), formed by PB2, PB1, and PA, encasing the termini of the viral genome (vRNA), itself coated with nucleoprotein (NP). **(C)** Organisation of the IAV genome, highlighting the major alternatively spliced segments, M and NS. **(D)** Overview of the viral replication cycle, focussing on the major RNA trafficking events. After endocytosis, vRNPs escape in the cytoplasm and are transported to the nucleus, where they can generate viral mRNAs and replicate new vRNA segments through a full-length positive sense intermediate (cRNA). The former are efficiently exported and translated into viral proteins, while the latter is trafficked to budding sites at the plasma membrane. Graphical representations of M1, M2, HA, NA, envelope, cell membrane, nucleus, arrows, and ribosomes are adapted from Servier medical art repository (https://smart.servier.com).

As shown in Fig 1, IAV is an enveloped, negative-sense, single-stranded, segmented RNA virus. At the core of the virion resides the genome, subdivided into eight separate RNA segments (vRNAs) ranging in size from ~900 to ~2300nt (Fig 1C), each encapsidated into viral ribonucleoparticles (vRNPs). Here, the vRNA is folded on itself, forming a panhandle structure in which both the 5′ and 3′ ends are secured inside the influenza RNA polymerase (or FluPol) [3], which is formed by three subunits: PB2, PB1, and PA. The rest of the vRNA is coated by several units of the viral nucleoprotein NP, each covering an average footprint of ~24nt (Fig 1B) [4]. The eight vRNPs sit at the centre of the viral particle in a 7+1 conformation, in which seven segments encircle the remaining molecule (reviewed in [5]). Other than viral proteins, virions have been found to also package a range of host proteins but their contribution to the viral physiology is unclear [6].

The viral replication cycle is represented in Fig 1D and it begins with the attachment of the virion to the cell surface, aided by the recognition of sialic acid glycoproteins on the host membrane by the viral HA [7]. Once attached, virions are endocytosed and travel towards the nucleus, where transcription and replication take place. First, the endocytosed vesicles undergo endosomal maturation, gradually lowering their internal pH. This triggers a cascade of structural changes in the virion that culminates in the fusion of the envelope with the endosomal membrane and the uncoating of vRNPs from the matrix (reviewed in [8,9]). The viral genome is then trafficked to the nucleus through the nuclear pore complex [10].

All viral transcription, encompassing mRNA production and genome replication is carried out in the nucleus by the influenza RNA polymerase. The regulation of FluPol, with its different activities and modalities, is reviewed in detail elsewhere [11–13]. Viral mRNA transcription is initiated by cap snatching: the FluPol targets actively transcribing host RNA polymerase II (RNAPII) complexes and binds the nascent capped transcripts, the PA subunit then cleaves the 5′ cap and residual nucleotides and utilises it as a primer to generate a viral transcript. Cap-snatching is an important way the virus ensures high rates of translation of its mRNAs. The majority of IAV transcripts are unspliced, with M and NS segments being alternatively spliced to minimally generate M1 or M2, and NS1 or NS2, respectively (Fig 1C, right) [14,15]. This is in contrast with the host, in which the vast majority of mRNAs are spliced, and splicing itself is an important step in quality control and nuclear export (as reviewed in [16]). Thus, influenza has evolved different strategies to ensure proper localisation of both unspliced and spliced transcripts, while trying to remain undetected by immune sensors.

During infection, with the accumulation of viral proteins, the FluPol switches modality and begins transcribing genome copies, first generating positive-sense intermediate products (cRNA). Both cRNAs and newly generated vRNAs are co-transcriptionally encapsidated into RNPs with many NP molecules and a single trimeric FluPol complex. The genome copies then undergo nuclear export via a chain of interactions between the vRNP, M1, and the CRM1 system [17–19]. Upon export, the individual segments associate with the host recycling endosome network for vesicular transport towards the plasma membrane (PM) [20–23]. This step is a crucial part of the replication cycle where segments are sorted for packaging, but the precise mechanisms that allow for vRNP trafficking, segment bundling, and virion assembly are still not fully elucidated.

The replicated vRNPs gather at specialised, virus-induced lipid raft domains enriched in cholesterol and viral glycoproteins [24]. Virion budding and release is a multi-step process involving the concerted action of several host and viral factors [20,25–28], and it is still incompletely understood.

## 2. Viral entry: The genome’s path to the nucleus

### 2.1. Entry and uncoating

**Fig 2:**
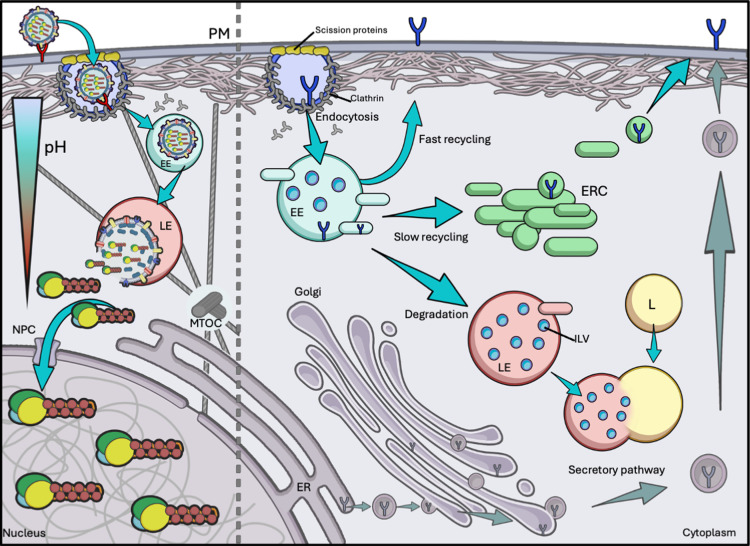
Viral entry and host endomembrane systems. On the right side, the major endomembrane systems of the host cell are represented. At the top, endocytosed material (e.g., signalling receptors, in blue) is trafficked to early endosomes (EE), where it can be sorted for fast (directly to the membrane) or slow (transiting through the endocytic recycling compartment, ERC) recycling, or for degradation. In the latter case, it is directed to late endosomes (LE) which then fuse with lysosomes (L). On the bottom, the secretory pathway involves protein production at the endoplasmic reticulum (ER) and trafficking through the Golgi apparatus to acquire post-translational modifications, before reaching the membrane. On the left side, endocytosed virions reach EE, and then LE. Acidification of the vesicle triggers vRNP release. The viral genome is subsequently imported into the nucleus. Graphical representations of cell membrane, nucleus, arrows, virion, actin, scission proteins, receptors, vesicles, ER Golgi, and NPC are adapted from Servier medical art repository (https://smart.servier.com).

Viral entry is a key step in host adaptation. At the host’s PM, the virion engages in transient interactions with the cell’s glycoproteins, scanning the surface for the appropriate sialylated receptor to trigger the start of the infection [29]. This is aided by the sialidase activity of NA cleaving unproductive HA bindings, as well as cooperative low-affinity binding events [30]. IAV appears to have evolved a few different strategies to enter the cell, with the most studied “canonical” route via clathrin-dependent, receptor-mediated endocytosis [31]. Upon entry, endocytosed virions are contained in lipid vesicles, and subsequently intersect the vesicle trafficking network (Fig 2, left).

The host’s vesicle trafficking apparatus is a sprawling network of interconnected, non-linear, highly regulated vesicular transport systems, fundamental for cell homeostasis and the regulation of responses to stimuli [32]. Fig 2, on the right, shows a simplified representation of these interconnected systems. Particularly important for IAV trafficking is the endocytic pathway, the branch overseeing the internalisation of cargo from the PM. Examples of cargo molecules are signalling receptors and ligands, nutrients, and membrane components. Endocytosed material can have very different fates, either being recycled to the PM (mainly) through the endosomal recycling compartment (ERC) or degraded via the endolysosomal pathway (reviewed in [32–34]). Particularly, several multiprotein sorting complexes in the early endosome (EE, also referred to as the sorting endosome) can recognise specific signals such as ubiquitination or sorting motifs, and direct the cargo towards the ERC, or degradation by confining it into intraluminal vesicles (ILVs). Due to the presence of ILVs, late endosomes (LE) are also referred to as multivesicular bodies. Endosomes are trafficked on actin filaments [35] at the cell periphery and then transported from the PM towards the perinuclear region on microtubules (MTs) [36]. Here they undergo maturation by acidification and fusion with vesicles from other parts of the network (thoroughly reviewed in [37]). Lastly, mature late endosomes fuse with lysosomes, allowing catabolic enzymes to degrade the remaining cargo [38].

Influenza virus has evolved sophisticated strategies to hitchhike on the vesicular transport network during entry towards the nucleus, virion assembly, and egress from the host cell. The virion is a metastable structure; on its own the virion is impermeable and tightly compacted. However, during entry and uncoating, a series of conformational changes in M2, M1, and HA triggers the fusion of the envelope with the endosomal membrane and the dissolution of the matrix, forcefully ejecting the vRNPs into the cytosol (Fig 2, left) [39–42]. These processes are generally difficult to study due to their very fast kinetics, with free vRNPs detected in the cytoplasm in a matter of minutes post-inoculation [43]. Important research on the pH requirements for infection and fusion demonstrated that fusion and uncoating are multi-step processes, dictated by the progressive acidification, and changes in ionic composition of the endosomal and virion lumens [39,44]. Further structure work highlighted the existence of several transition states in HA before and during membrane fusion, some of which are reversible, and regulated by pH [45,46]. The HA-mediated fusion of the envelope with the endosomal membrane is necessary but not sufficient to release the genomic segments from the virion. vRNPs are connected to the envelope by a strong interaction between NP and M1 in the matrix, itself bound to the luminal tails of HA and NA [47–49]. The proton channel M2 plays a fundamental role in coupling the maturation of the endosome with the acidification of the virion lumen, and it is itself activated by this acidic environment [50,51]. Indeed, the low pH-dependent conformational changes needed for vRNP release can be blocked by inhibiting M2 [52]. Uncoating, the essential step of M1 detaching from the genome, starts at the slightly acidic pH (~6.5) of the early endosome, where the matrix is primed, and its integrity is softened [44]. This allows its dissociation at the low pH (~5.5) of the LE, concurrently with HA-dependent membrane fusion. Earlier research describing the overexpression of M1 in cells prior to infection, found that vRNPs released into the cytosol were prevented from entering the nucleus, with further experimentation validating that this was due to binding of the uncoated vRNPs by host-produced M1 [53]. The same group also found that even microinjection of M1-associated vRNPs prevented nuclear localisation of genomic segments, further substantiating M1 detachment as a vital step in vRNP nuclear import [54].

Following endosomal fusion, viral components gain access to the cytoplasm, but the uncoating process is not yet complete. Further host factors are involved in removing vRNPs from the virion, but their precise mechanism of action is mostly unclear. An interesting example is the histone deacetylase HDAC6. Contrary to what its name suggests, this enzyme is predominantly localised in the cytoplasm, where it acts on MTs [55], HSP90 [56], and RIG-I [57], among others. The deacetylase function of HDAC6 appears to be antiviral, with a study reporting increased infectious viral production following HDAC6 inhibition, also showing an increase in HA at the PM [58]. Moreover, the HDAC6-mediated deacetylation of RIG-I activates the receptor, facilitating the recognition of viral RNA [57]. However, HDAC6 also encodes for a zinc finger domain capable of binding ubiquitin (Ub) chains [59]. In healthy cells, HDAC6 is involved in the recognition of polyubiquitinated misfolded proteins, and their recruitment to the aggresome [60]. The aggresome is a perinuclear structure aggregating misfolded proteins and devolved to their clearance. The aggresome contains several chaperones and components of the proteasome. HDAC6 in this context works as an adaptor connecting misfolded proteins and MTs by its association with the dynein motor. Importantly, free ubiquitin chains have been reported as virion components by microscopy [61] and proteomic [62] approaches. IAVs have evolved to exploit the misfolded protein response to recruit HDAC6 and the cytoskeleton to uncoating virions, enhancing the extraction of vRNPs thanks to the pulling force of dynein on MTs close to the nucleus [61]. The apparent contradiction of pro- and anti-viral functions of HDAC6 still hasn’t been entirely cleared, and different groups reported contrasting effects of HDAC6 depletion on viral replication in mouse models [61,63]. A further contribution to uncoating is carried out by importin-β (TNPO1) which acts as a chaperone by recognising a nuclear localisation signal (NLS) on M1 and separating it from vRNPs, and siRNA-mediated depletion of this factor severely reduced viral infectivity [64]. Uncoating is a fundamental process that allows vRNPs to enter the nucleus, but the role of individual host factors and pathways implicated in this process still isn’t fully elucidated.

### 2.2. Nuclear translocation

In order to successfully initiate the infection, vRNPs must reach the host nucleus. However, it has remained unclear for some time whether all eight vRNPs collectively migrate into the nucleus upon uncoating, or whether they traffic individually. Visualisation of quantum dot conjugated vRNPs has provided strong evidence for the latter scenario, with clear imaging of two vRNPs being released separately from the virion into the cytosol [65]. Furthermore, this same research demonstrates the translocation of individual vRNPs, followed by a random distribution of these complexes in the nucleus upon trafficking, though further validation of this process by other means would be welcomed [65].

**Fig 3:**
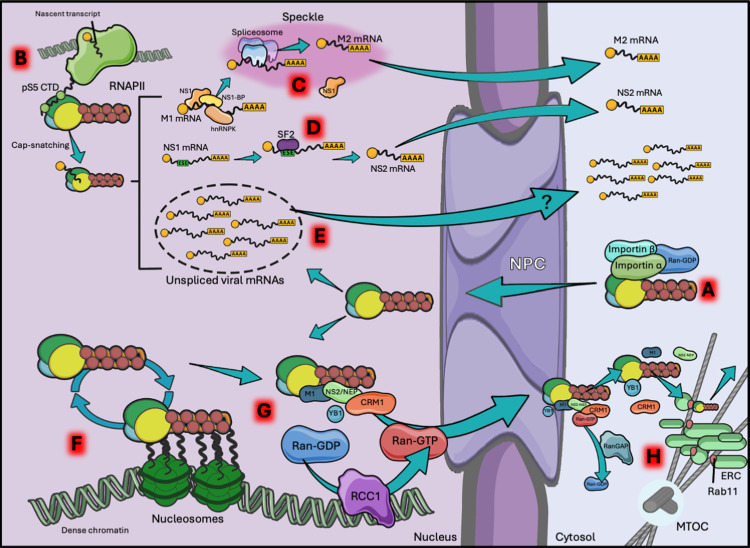
Trafficking through the nuclear pore. **(A)** In the cytosol, NLSs on the vRNP are targeted by the importin system for translocation through the NPC. **(B)** In the nucleus, vRNPs localise to sites of active host transcription by binding to the RNA polymerase II (RNAPII) C terminal domain (CTD), and perform cap snatching. **(C)** M1 mRNA is spliced into M2 by trafficking to the nuclear speckle by interaction with viral NS1, and host NS1-BP and hnRNPK. **(D)** NS1 mRNA is spliced into NS2/NEP in the nucleoplasm by the concerted action of several splicing factors, initiated by binding of SF2 to an exonic splicing enhancer (ESE). Both NS2 and M2 are translocated to the cytosol at the end of the splicing process. **(E)** The precise mechanisms with which unspliced viral mRNAs reach the cytosol are currently unclear. **(F)** Viral genome replication is segregated to the dense chromatin, by interaction of vRNPs with nucleosomes. **(G)** Proximity to the chromatin-associated RCC1 provides newly replicated vRNPs with privileged access to the export machinery. **(H)** After dissociation of the export machinery, vRNPs are loaded (possibly by YB-1) on ERC vesicles at the MTOC. Graphical representations of the NPC, nuclear membrane, microtubulses, vesicles, M1, arrows, DNA, host proteins, and the spliceosome are adapted from Servier medical art repository (https://smart.servier.com).

The nucleus is separated from the cytoplasm by a double membrane with the only entry points being the nuclear pores. These complex multi-subunit structures block the passage of cargo larger than 9 nm so most macromolecules can only enter by active transport (nucleus permeability has been extensively reviewed elsewhere [66]). Briefly, the direction of traffic through the pore is regulated by the GTPase Ran. Ran and all related GTPases have a very low efficiency of GTP hydrolysis, mostly relying on cofactors to induce the reaction or the exchange of the bound nucleotide. In the case of Ran, the guanine exchange factor RCC1 binds chromatin and is thus exclusively nuclear, while the GTPase activating protein (GAP) RANGAP is cytoplasmic. This creates a gradient in which Ran binds GTP in the nucleus and GDP in the cytoplasm. Moreover, three classes of transporters are identified by their directionality: importins that traffic towards the nucleus, exportins leaving it, and the bidirectional biportins. Proteins containing NLSs are recruited by importins, dock at the NPC, and proceed into the nucleoplasm. There, Ran-GTP associates with the importin, dissociating the cargo. Proteins to be exported from the nucleus harbour nuclear export signals and are recognised by exportins. These carriers bind with cargo and Ran-GTP cooperatively, creating ternary complexes that travel through the pore. In the cytosol RANGAP induces GTP hydrolysis, releasing the cargo (reviewed in [67]). Although all protein components of the vRNP harbour NLSs [68–73], the biggest contributor to vRNP import in the nucleus is NP, which carries three independent NLSs, normally occluded by M1 binding and described above. In particular, the unconventional NLS in an unstructured domain at the N terminus of the protein seems to be the most important for genomic localisation [70]. Once the virion dissociates, and vRNPs undergo uncoating, the matrix no longer masks the NLSs, and the particle is imported very efficiently by interaction with several importins, as shown in Fig 3A [74,75]. Interestingly, it is now clear that adaption to host-specific nuclear import machinery is another barrier to interspecies transmission of IAV. Gabriel and colleagues (2008) initially described mouse adaptive mutations in an avian H7N7 virus, namely PB2 D701N and NP N319K, and demonstrated enhanced binding for mammalian importin-α1 [75]. Further work from the same group demonstrated that, while avian- and mammalian-adapted IAVs are equally dependent on importin-α1, loss of importin-α3 was found to greatly reduce the replication of avian influenza, while loss of importin-α7 negatively affected the replication of human IAVs [76]. The authors proceeded to highlight the potential importance of importin-α3 in the early stages of cross-species adaption, due to the very high conservation rate of importin-α3 (>99%) between avian and mammalian species [76]. However, all this work does not account for the possibility that importins may play secondary roles during viral replication, and therefore their direct importance for vRNP nuclear import alone is yet to be proven.

Once in the nucleus, vRNPs target the host chromatin by interaction of NP with the tails of all core histones (Fig 3F), in order to gain proximity to the cellular transcription machinery [77]. To perform cap snatching and initiate viral transcription, the viral polymerase requires close contact with the actively transcribing host RNAPII. This is achieved by specific interactions between PA (particularly the PA-C domain) and the C terminal domain (CTD) of the catalytic subunit of RNAPII (RPB1) [78]. This domain is characterised by multiple repetitions of a heptad motif YSPTSPS, and its phosphorylation state is crucial to regulating the transcription state of the polymerase (Fig 3B). Interestingly, the interaction with the vRNP is only possible when the heptad is phosphorylated on its 5^th^ position (pS5), the signature for RNAPII initiating transcription [79,80]. Thus, PA is positioned close to nascent transcripts and able to cap-snatch. Moreover, the vRNP directly interacts with the exosome, a multi-subunit complex with exonuclease activity, which is responsible for the co-transcriptional degradation of many regulatory RNAs, suggesting that the FluPol could cap-snatch and recycle these transcripts [81]. Specifically, Koppstein and colleagues employed high-throughput sequencing of influenza mRNA 5′-termini to determine the identity of the cap-snatching repertoire of IAV, with small nuclear RNAs and small nucleolar RNAs providing the bulk of capped primers [82].

## 3. Viral mRNA trafficking, the complication for nuclear-replicating RNA viruses

One of the main hurdles viruses need to overcome to replicate efficiently is the innate immune system, and in particular the self versus non-self detection apparatus (comprehensively reviewed in [83]). In order for transcripts to be translated efficiently and not elicit an immune response, viral transcripts need to appear similar to host mRNAs. Different cytosolic RNA viruses approach this problem in a variety of ways, with some encoding their own RNA-modifying enzymes to introduce the signature mRNA caps [84–88], others employing cap proteins [89], and some actively recruiting the ribosome to avoid cap recognition entirely [90]. Influenza, on the other hand, employs a very different strategy. By localising to the nucleus, IAV has access to the full repertoire of mRNA processing machinery, and thus is able to generate viral transcripts that are virtually identical to host mRNAs. However, nuclear transcription introduces a new problem; exporting the transcripts out of the nucleus and to the translation machinery.

For the majority of host mRNAs, transcription, maturation, and export are strictly linked, with constant regulation happening around splicing [16]. During infection, IAV generates at least 10 transcripts: six are entirely intronless (PB2, PB1, PA, HA, NA, and NP), two contain an intron and can be translated without processing (NS1 and M1), and the last two are spliced variants of the latter couple (NS2 and M2). Given the heterogeneity of maturation requirements, IAV utilises different pathways for different mRNAs, but the precise mechanisms for each type of viral mRNA remain elusive.

Nuclear speckles are subnuclear biocondensates present in host cells and act as reservoirs of splicing machinery (extensively reviewed in [91]). Generally, most cellular splicing occurs co-transcriptionally, with the spliceosome being recruited from speckles to pre-mRNAs during polymerase stalling [92–94]. However, this modality is not available to viral mRNAs transcribed by the FluPol. Recently proven by the Fontoura group, the M1 pre-mRNA is instead recruited in the nucleoplasm and transported to nuclear speckles where it is spliced and marked for export [95,96]. Specifically, NS1 targets the mRNA and associates with the adaptor NS1 binding protein (NS1-BP), and the heterogeneous nuclear ribonucleoprotein K (hnRNP K) which brings the transcript to the U1 spliceosomal snRNP residing in the speckle, kickstarting the process (Fig 3C). Further validation of this mechanism by gene manipulation found that knockdown of either NS1-BP or hnRNP K significantly reduced the abundance of M2 mRNA when compared to the unspliced M1 transcript [96]. The use of NS1 for promoting M2 expression is an interesting example of temporal regulation of viral gene expression, fine-tuning the relative amounts of M1 and M2 during infection. NS splicing, on the other hand, does not rely on speckles, instead being directed to and spliced in the nucleoplasm [97,98]. NS1 pre-mRNA carries a weak splicing site and an exonic splicing enhancer (ESE) that is recognised by the splicing factor SF2 (Fig 3D) [99]. Huang and colleagues (2017) uncovered that the ESE is a site of potential adaptive mutation, with the G540A nucleotide polymorphism found in an H7N9 avian virus shown to contribute to enhanced replicative fitness in mammalian cells, without affecting fitness in avian cells [99].

mRNA export from the nucleus is mediated by two main transporters: chromosomal maintenance 1 (CRM1) and nuclear RNA export factor 1—nuclear transport factor 2-like export factor 1 (NXF1-NXT1). The former being the predominant generalised exportin, and the latter a specialised RNA carrier. To date, there is no evidence suggesting CRM1 is involved in the export of IAV mRNAs, with many viral mRNAs seemingly reliant on NXF1 [100]. Generally, host mRNAs interact with the transcription and export complex (TREX) during splicing, in order to contact NXF1-NXT1 and translocate across the NPC. However, the evidence around the requirement for TREX components as well as NXF1 is uncertain, with perhaps cell line- or strain-specific effects at play [100,101]. Additionally, it is likely that other adaptor proteins could be selectively involved in trafficking IAV mRNAs, especially for unspliced transcripts. Intriguing work from Bhat and colleagues suggests that a key contributor to IAV mRNA export is germinal-centre-associated nuclear protein (GANP), a subunit of the TREX-2 complex [102]. This carrier is involved in the transport of a subset of host mRNAs that share some common features with IAV mRNAs such as low exon count and exon length. GANP would recruit IAV transcripts into the TREX-2 complex and to NXF1. The composition of the NPC is also important, with the nucleoporin TPR acting as a fundamental docking element for GANP. These data were validated through the manipulation in expression of GANP, TPR and the TREX-2 component PCID2 using an auxin-induced degron system [102]. This allowed authors to rapidly degrade key host proteins after infection had already commenced, to minimise potential cytotoxicity issues that may arise from manipulation of host nuclear export machinery. In each instance, smFISH followed by quantification of fluorescence intensity clearly demonstrated a significant increase in nuclear localisation of HA, M, and NS mRNAs [102].

Another piece of evidence towards IAV employing non-canonical export pathways is the recent discovery by our group that during infection, influenza mRNAs were specifically recruited by MKRN2, with this association depending on cis-acting packaging sequences on the transcript [103]. MKRN2 is an understudied RNA binding protein (RBP), known to mediate the nucleocytoplasmic localisation of a subset of host mRNAs [104]. Depletion of this RBP led to a specific depletion of influenza transcripts in the cytosol, accompanied by a reduction in viral replication. Overall, it is apparent that more research into alternative mRNA export pathways is necessary to truly understand how IAV specifically controls the localisation of its transcripts (Fig 3E).

## 4. Genome egress: The odyssey of the vRNP

### 4.1. Nuclear export

Following viral mRNA export and protein production, newly synthesised vRNP components are imported to the nucleus where they kickstart viral replication. IAV genome replication, as well as the modalities and host factors involved in viral transcription, have been investigated extensively in the past decades. However, the issue of subnuclear localisation of the replicating vRNP has not been studied in as great detail. IAV infection induces strong perturbations in the nuclear architecture, with viral elements interacting with, displacing, or recruiting some of the most important subnuclear domains [95,105–107]. An interesting example is IAV’s interaction with nucleoli. Several works detail the requirement of nucleolar proteins for viral replication as well as the direct interaction of NP and PB1 with the nucleolar marker nucleolin, leading to its relocalisation to the nuclear periphery [106]. This interaction in particular seems to be important for vRNP trafficking, with knockdown of nucleolin in infected cells previously shown to drastically alter the nucleocytoplasmic distribution of vRNPs. With significantly more vRNA in the nucleus, viral production was also found to be negatively affected [106]. It was proposed that the role of nucleolin in vRNA nuclear export may be in guiding vRNPs to dense chromatin regions. Here, vRNPs gain privileged access to the nuclear export machinery since it is the site of Ran-GTP regeneration by RCC1 (Fig 3F) [107,108]. Newly replicated vRNPs in dense chromatin regions form a “daisy chain” by binding to M1, which then contacts NS2 (a.k.a. nuclear export protein, NEP) [109] bridging the interaction to CRM1 and Ran-GTP (Fig 3G). The carrier complex is then translocated through the NPC and GTP hydrolysis dissociates the cargo (Fig 3H). Moreover, some evidence points also to redundant passive export of vRNPs following caspase-mediated enlargement of the NPC [110].

### 4.2. Cytosolic trafficking of vRNPs and ERC remodelling

vRNP trafficking in the cytoplasm is a very active and prolific field of IAV research, since a better understanding of this step could lead to breakthroughs on reassortment and genetic shift.

**Fig 4:**
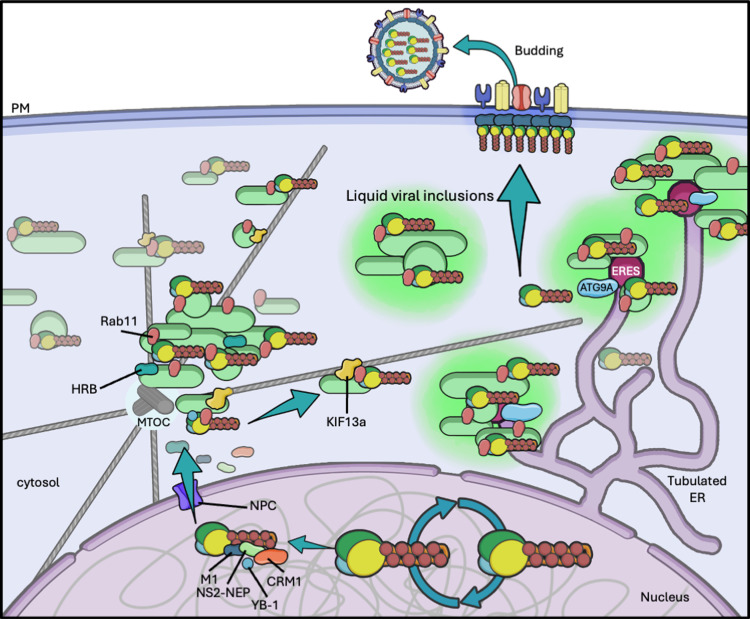
Proposed model for cytosolic vRNP trafficking. After viral genome replication, vRNPs are exported to the cytosol where they are loaded onto Rab11-positive vesicles at the MTOC. Extensive remodelling of the endomembrane systems of the cells upon infection produces ER tubulation and enlarged Rab11-/vRNP-positive puncta. MT-associated vRNP-clad Rab11+ vesicles travel towards the plasma membrane by action of molecular motors as KIF13a, and reach ER exit sites (ERES) where they are unloaded by action of ATG9A. ERES are also sites of viral inclusion phase separation. The means of vRNP delivery to budding sites is still incompletely elucidated. Graphical representations of cell membrane, nucleus, virion, microtubules, host proteins, NPC, and arrows are adapted from Servier medical art repository (https://smart.servier.com).

Several seminal papers in the last decade described the importance of the recycling network, and in particular the marker GTPase Rab11, in the cytosolic trafficking of vRNPs [21–23,111–113]. This is in line with a mounting body of literature detailing the role of this protein and the broader recycling pathway in the egress of a multitude of viral pathogens [114–117], and its importance in the replication of numerous bacteria [118–120]. Fig 4 summarises our current understanding of this process. Newly synthesised vRNPs quickly accumulate near the microtubule organising centre (MTOC) upon nuclear egress, colocalising with Rab11 [21–23]. The cellular Y-box-binding protein 1 (YB-1) is an important regulator of host mRNPs and a component of key stress-related organelles such as stress granules and P-bodies (reviewed in [121]). It has been proposed that YB-1 undergoes relocalisation to the nucleus upon IAV infection where it associates with vRNPs during export [122]. In the cytoplasm, it colocalises with vRNPs and Rab11 at the MTOC, suggesting a role for YB-1 in delivering viral particles to the ERC. However, in the absence of any published data describing the effect of YB-1 loss on the production of progeny virions, it remains unclear whether this proposed interaction is an essential step in vRNP trafficking or simply a consequence of trafficking on Rab11-positive vesicles. Some studies also suggest the HIV rev binding protein (HRB) could be involved in this step, or possibly beyond, since its RNAi-mediated ablation leads to the arrest of vRNP trafficking in the cytosol [123].

Some calculations suggest that healthy cells endocytose their entire surface at least once an hour, and thus require robust recycling pathways to retrieve both lipids and membrane proteins to avoid waste (extensively discussed elsewhere [124]). Cargo sorting in the early endosome can lead to the formation of tubular vesicles containing cargo to be recycled. These undergo maturation, losing the early endosomal Rabs and acquiring the recycling-specific Rab11, as well as Rab11-family interaction partners (FIPs), and cluster around the MTOC (in most cell types) before travelling towards the membrane. Interestingly, some evidence suggests that ERC tubulation from the early endosome could involve contact with the endoplasmic reticulum [125]. Moreover, Häsler and colleagues recently found that certain cargo in the ER secretory pathway transits through the ERC on the way to the PM, highlighting the extremely interconnected nature of these endomembrane systems [126].

Given the extensive colocalisation of vRNPs and Rab11 during egress, it has been long speculated that IAV could be hijacking the recycling pathway to be efficiently transported to the PM. In this model, vRNP-clad ERC vesicles would move along MTs, perhaps colliding along the way and fusing, bringing together all eight segments, until they reach the budding sites on the PM.

Several points of evidence, however, indicate that a more complex scenario may be at play. First, contrary to what might be expected if it were the final carrier to the PM, Rab11 itself is not found in virions [6,127], or even at the PM [23]. Nevertheless, these data could still be compatible with direct Rab11-mediated vRNP transport to the membrane. In this instance, Rab11 vesicles coated with vRNPs would touch the PM, unload the genomic segments, and quickly detach, never fully fusing with the membrane. In any case, this remains an open question that requires further investigation. Secondly, it is now evident that influenza infection deeply perturbs the physiological recycling compartment, altering its morphology, composition, and function. In healthy cells, Rab11 is localised throughout the cytoplasm in a dispersed pattern and accumulates at the MTOC. Upon infection, Rab11 foci in the cytoplasm and the MTOC accumulation both enlarge and colocalise with vRNPs [21–23]. The recycling activity itself is largely abrogated, with some cargo like the transferrin receptor confined to the MTOC upon infection [128]. The reduced function of the ERC could be in part explained by the decreased interaction of Rab11 and Rab11-FIPs, due to the competition of vRNPs for binding site [128]. Correlative light and electron microscopy experiments found profound changes in the ultrastructure of the compartment, which is defined by the clustering of vesicles decorated with vRNPs dubbed irregularly coated vesicles (ICVs), as well as double-membrane structures, perhaps indicative of the involvement of the lysosomal pathway [111]. The ER is also remodelled upon infection, forming tubular structures [111]. In particular, ER exit sites are seemingly involved in seeding Rab11 foci [129]. Here, the autophagy-related gene A9 (ATG9A) is thought to unload vRNP-clad Rab11-positive vesicles from MTs, promoting the formation of characteristic round viral inclusions [125]. Counter to this, the loss of ATG9A was shown to induce the formation of diffuse tubular viral inclusions, and significantly reducing viral titres [130]. Dissecting the complexity of the remodelled system is of great importance to deconvolve the modified pathways forged by IAV for vRNP trafficking. Recent results from our group, suggest the involvement of the transmembrane protein Myoferlin (MYOF) in this process, as well as its inclusion in ICVs. Our evidence indicates that loss of MYOF expression reduces infectious virus production and that the co-factor EHD2 may be recruited to these ICVs, further expanding the elusive list of host factors that form part of this pathway [131].

Another interesting point of debate in the community is the involvement of the cytoskeleton in vRNP transport. During viral entry, virions follow the endocytic pathway and exploit the actin network first and then MTs to reach the nuclear periphery [132,133]. However, the process is more complicated during egress, largely due to the seemingly drastic reorganisation of vesicular systems in the infected cell. In physiological conditions, recycling largely depends on movement on MTs, with some Rab11 effectors providing tethering and motor function, such as the motor protein KIF13A. This kinesin-3 protein, important for the biogenesis of the ERC [134], was found to efficiently transport vRNP-clad Rab11 vesicles on MTs towards the cell periphery [135]. This role was further supported by KIF13A depletion, which was found to reduce viral titres through reduction of vRNPs accumulating at the cell surface, while levels of HA and M2 remained unaltered [135]. Microtubule disruption, for example by treatment with the MT depolarising drug nocodazole, altered the localisation and movement kinetics of vRNPs [21], decreased vRNP colocalisation with Rab11 [136], and severely impacted fusion-fission events of liquid viral inclusions [129]. Despite this evidence towards the important role of MTs in vRNP trafficking, nocodazole treatment only modestly impacted the production of infectious virions [21,129]. Thus, it is very likely that MT transport is only part of a redundant mechanism, and IAV employs several strategies at once to ship its genomic segments to the membrane. Several works suggest that the actin cytoskeleton might work in concert with MTs to direct vRNP transport [137,138]. Live-cell imaging experiments allowed the tracking of single particles and the characterisation of their movement. Upon treatment with cytoskeleton depolarising drugs (nocodazole for MTs and cytochalasin D for actin), a clear synergy between the two modes of transport was detected [137,138]. Furthermore, the actin cytoskeleton is important in the cell-to-cell spread of vRNPs by tunnelling nanotubes, cellular processes connecting the cytosol of neighbouring cells [139,140]. These findings highlight the interconnectivity and redundancy between cellular macromolecule transport systems. Thus, it is possible that different branches of the cytoskeleton could have primary dedicated functions in vRNP trafficking (e.g. MTs in vesicular transport of newly replicated vRNPs in the cytosol), but that these can be supplied by other means, albeit with perhaps lower efficiency.

### 4.3. The problem of bundling

The segmented nature of the IAV genome confers the strategic evolutionary advantage of easy recombination by exchanging vRNPs between different coinfecting strains, greatly boosting the genetic pool of the virus. However, by having its genome confined to eight separate molecules, each indispensable for infection, IAV requires efficient selective bundling (the gathering of each individual genomic segment) and packaging (the introduction of the segment bundles in the virion) of vRNPs to generate competent virions. Throughout the years, two main models have been proposed for IAV bundling: random and selective. The first posits that incorporation in virions would be stochastic, while the latter predicts that specific interactions between segments would drive the bundling of the full set. IAV infection is not wholly efficient, and it is thought that often more than one incoming virion is required to generate a productive infection [141], lending credence to the random incorporation hypothesis. However, although different types of packaging defects have been detected, it is also likely that inefficiencies could derive from failures of uncoating/transport to the nucleus or mutated/defective vRNPs. Moreover, a purely random bundling process would lead to the generation of an overwhelming (>6000) number of set combinations, of which only a handful would be competent for infection. Currently, the selective model is the most supported by evidence, but there are still interesting open questions regarding the specific interactions guiding the process, as well as where these would take place [142].

**Fig 5:**
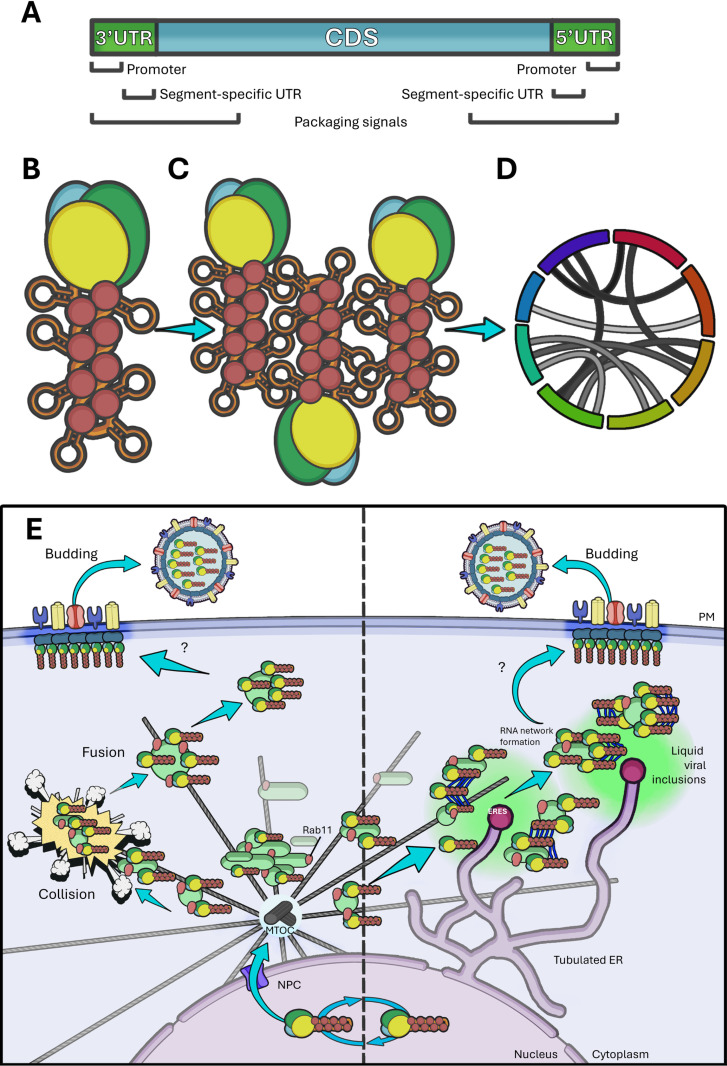
The problem of bundling. **(A)** Schematic representation of cis-acting packaging and bundling sequences in IAV RNA segments. Adapted from Noda et al. [93]. **(B)** Representation of secondary structures on the vRNA. **(C)** Model of inter-segment RNA-RNA interactions. **(D)** Inter-segment interaction maps have been generated to explore the interaction networks established during infection. **(E)** Representation of the main models being investigated to explain the formation of genomic bundles: on the right, the dispersed model argues that collisions between vRNP-containing vesicles could lead to the formation of vesicles containing multiple segments, facilitating bundling; while on the left, the compartmentalised model sees the formation of bundling hot spots (for example by phase separation) in which several vesicles can exchange segments, then the bundles would be able to progress to the membrane. Graphical representations of cell membrane, nucleus, virion, microtubules, host proteins, NPC, vesicles, and arrows are adapted from Servier medical art repository (https://smart.servier.com).

In order to achieve specific packaging of each different segment, sequence-level information is needed. Each segment contains common terminal non-coding sequences involved in packaging, but it is evident that individual segment-specific sequences are also involved (Fig 5A) (this has been thoroughly reviewed elsewhere [142]). A breakthrough in this field came from the Lakdawala group by employing high-throughput sequencing of RNA isolated by crosslinking and immunoprecipitation (HITS-CLIP) [143]. They found that NP coverage of the vRNA is incomplete, and preferentially binds G-rich and U-poor regions. Other similar results led to speculation that vRNA stretches not involved in NP binding could be free to form secondary/tertiary structures (Fig 5B) and even be available for inter-segment interactions (Fig 5C) [144,145]. Several maps of IAV vRNA interaction networks have been made, using different crosslinking RNA sequencing methods, with contrasting results [146–150]. It is evident that these interactions are generally not well-conserved, can vary greatly among strains, can be altered by differences in NP protein, and are highly plastic (Fig 5D). Several interactions have been mapped to the untranslated regions of each segment (UTR), where the packaging signals reside, but also span the coding sequences [5,142]. The specific contribution that each of these provides to the efficiency, selectivity, and robustness of the process is still unknown, but their importance is widely recognised.

Single-molecule fluorescent in situ hybridisation (smFISH) allowed for the imaging of multiple vRNA segments at once in infected cells and several reports indicated that colocalisation of different segments increased proportionally to the distance from the nucleus, suggesting that genomic bundles could be formed during transport [151–154]. This culminated in the dispersed model of bundling (Fig 5E, left), which predicts that random collisions between Rab11 vesicles carrying vRNPs would lead to intersegmental interactions and bundle formation. Conversely, the more recent compartmentalised model of bundling (Fig 5E, right) suggests that the bulk of bundling would occur at Rab11 vesicle clusters arising from ERC remodelling, facilitated by the high local concentration of vRNPs. An element supporting this model is the important discovery, by Alenquer and colleagues in the Amorim lab, that virus-induced Rab11/vRNP inclusions in the cytoplasm appear to be biocondensates [129]. Biocondensates are membraneless compartments with liquid properties defined by multivalent weak interactions between their constituents (reviewed in [155]), and are increasingly being studied in the context of viral infections [156–158] (reviewed in [159]). In this context, vRNPs (both free and on Rab11 vesicles) could come into contact and freely explore the interaction space until bundles are formed, which might have a lower affinity for the condensate, and are shipped to the PM. Also, the multitude of highly plastic vRNA-vRNA interactions detected by crosslink sequencing could play a role in creating the conditions for phase separation.

Although the final carrier of vRNPs to the PM is still unclear, once the segments/bundles reach the PM they are recruited to specialised budding sites on lipid rafts by M1 via an M1–NP interaction, where virion proteins are clustered, and assemble into growing structures on-site [160,161]. While a number of host and viral factors have been shown to contribute to membrane deformation and ultimately virion budding, M1 appears to be the main driver of virus budding, with expression of M1 alone previously shown to induce the production of vesicular particles [162,163]. The clustering of glycoproteins, as well as the scaffolding of matrix protein, contribute to shaping the membrane environment. The host recycling system, and in particular Rab11, plays a role in orchestrating budding [20]. However, contrary to many other enveloped viruses (reviewed in [164]), the process does not rely on the recycling-derived endosomal sorting complexes required for transport machinery for membrane scission [25,26]. Instead, it appears that an amphipathic helix in the viral M2 localised in the bud neck may curve and destabilise the bilayer leading to membrane scission [27,28], though findings contrary to these data dispute this assertion [165]. The viral NA is also involved in the budding process by releasing the emerging virions from the sialylated glycoproteins on the membrane of producer cells [166].

## 5. Concluding remarks

Viruses are molecular machines that deliver exogenous genetic information to subvert the cell. As such, deciphering the host intracellular trafficking mechanisms and the ways in which they can be exploited during infection could provide important insights to combat broad classes of pathogens. For example, several reports have highlighted the importance of the Rab11 recycling endosome in the replication cycle of clinically important human pathogens including paramyxoviruses viruses [167,168], coronaviruses [114], hantaviruses [115], and retroviruses [169], in addition to some bacteria such as Salmonella spp [170] and Shigella spp (reviewed in [171]). In some cases, the vesicular network is exploited to transport viral genomes towards the membrane, in others to properly localise structural proteins to budding sites, or even to broadly escape the endocytic system. The control of RNA localisation in the cell is so important that even RNA viruses replicating in the cytosol often engage with components of the NPC or RNA exportins to block host mRNA export, contributing to host shutoff (reviewed in [172]).

IAV, a nuclear-replicating RNA virus, is an ideal model system to investigate host-pathogen interactions in RNA trafficking, given the breadth of the host transport pathways it intersects, hijacks, and disrupts to ensure its replication. In the decades that IAV intracellular trafficking has been investigated, many of the principal actors involved have been identified, as well as the major pathways that are employed. However, several questions still remain, which we detail in the next section, and more work is required to investigate the precise mechanisms that underlie many key steps.

IAV infection starts with HA engaging with sialylated receptors on the PM. However, a lot of research is currently underway to elucidate the presence and tissue distribution of particular sialic acid configurations in different species, an important area of study further emphasised by the recent spillover of H5N1 avian influenza into bovine mammary glands [173,174]. Additional research priorities to better understand initial infection include the importance of secondary receptors, and the contribution of clathrin-independent endocytosis to infection, particularly for what concerns filamentous clinical strains.

After endocytosis and late endosome maturation, vRNPs are uncoated and released in the cytoplasm. Here, though there is growing evidence for dissociation into individual vRNPs prior to nuclear translocation [65], it is still yet to be validated whether this dissociation occurs before or after nuclear import. Additionally, how the recruitment of importins is coupled with uncoating still remains uncertain, while there is mounting research demonstrating how host-adaption is driven by the exploitation of different importins between species.

Nuclear localisation of vRNPs is also being studied, with open questions regarding the involvement of the nucleoli, speckles, promyelocytic leukaemia bodies, and chromatin. During infection, the nucleus is transformed into a viral replication factory, so it stands to reason that all major nuclear systems would be affected. IAV is very competent at host shutoff, with several measures stopping cellular gene expression at all steps (the discussion of which is outside the scope of this review, and more thoroughly discussed in [175]), including mRNA export [176]. In this regard, it must strike a balance to maintain efficient viral translation by selectively translocating viral transcripts. IAV could be exploiting specialised cellular machinery involved in the processing of short/unspliced transcripts or coaxing other undiscovered adaptors or transporters.

In the last decade, important advancements in light and electron microscopy, as well as high throughput RNA sequencing have allowed for very in-depth studies of IAV trafficking in the cytosol and bundling, but many unknowns still linger. It is apparent that the whole endomembrane system is deeply perturbed during infection, but more research is needed to dissect the contribution of each compartment, particularly the modified ER, as well as how the remodelling is accomplished, and whether a final carrier of vRNPs to the membrane exists. Bundling also presents a very interesting challenge: segment selection appears to be very efficient and specific, but segment-segment interactions are dynamic and poorly conserved, suggesting the existence of other quality control mechanisms.

Overall, while the field has made significant progress in better understanding the trafficking network employed by IAV RNA during each stage of infection, through the development of new imaging and sequencing techniques, there remain many outstanding questions. Further exploration of this area could yield important insights into the replication cycle of numerous viral and non-viral pathogens and a keener understanding of overall cellular RNA localisation, while also providing a shortlist of essential host factors that may be targeted therapeutically to interrupt and halt viral infection.

## 6. Outstanding questions

- Do vRNPs dissociate into individual complexes during uncoating prior to nuclear import, or remain as a single large complex?
- What is the direct role of individual importin-α proteins in cross-species vRNP nuclear import, and how are they recruited to vRNPs upon uncoating?
- Where exactly do vRNPs localise in the nucleus and what is the involvement of various subnuclear compartments?
- Which host factors aid mRNPs in nuclear export while suppressing the export of host mRNAs?
- What is the exact role of each branch of the cytoskeleton in the trafficking of vRNPs?
- How do vRNPs selectively bundle to generate replication-competent progeny virions?
- Is Rab11, or an as yet undiscovered host factor, the final carrier for vRNPs at the plasma membrane prior to packaging?
- How is influenza virus adapted to exploit different trafficking machinery/pathways that may be encountered across diverse host species?
